# Assessement of burnout among high school teachers during the COVID-19 pandemic in Sfax, Tunisia

**DOI:** 10.1192/j.eurpsy.2022.1313

**Published:** 2022-09-01

**Authors:** N. Regaieg, L. Zouari, Y. Mejdoub, N. Smaoui, S. Omri, R. Feki, I. Gassara, N. Charfi, J. Ben Thabet, M. Maalej, M. Maalej

**Affiliations:** 1 Hedi Chaker University Hospital, Psychiatry C, Sfax, Tunisia; 2 Hedi Chaker university hospital, Epidemiology, Sfax, Tunisia

**Keywords:** Covid-19 pandemic, high school teachers, burnout

## Abstract

**Introduction:**

Facing educational difficulties related to COVID-19, some teachers can no longer adapt, making them potential candidates for burnout.

**Objectives:**

We aimed to assess burnout among high school teachers during the COVID-19 pandemic and to determine its prevalence and factors associated with it.

**Methods:**

We conducted a cross-sectional, descriptive and analytical study, carried out on google drive in May 2021, and relating to 97 Tunisian junior and secondary school teachers from the Sfax region. Burnout was evaluated by the Burnout Measure Short version (BMS-10).

**Results:**

The sex-ratio (M/F) of our population was 0.32 and the average age was 44.23 ± 7.81 years old. The labor load was low, medium and high in respectively 1.4%, 57.6% and 41% of cases. Almost a third of participants (30.6%) reported a low satisfaction with working conditions. The average BMS score was 40.19 ± 13.98. According to the BMS scores, 36.1% of teachers had a very low to a low degree of burnout, 23.6% had burnout while 40.3% had a high to very high degree of exposure to burnout. Furthermore, the BMS score was associated with the female gender (p=0.002), sleep disturbances (p<0.001), suicidal thoughts (p<0.001) and with a medium to a high labor load (p=0.045).

**Conclusions:**

In this study, Tunisian high school teachers in times of COVID-19 reported a high burnout rate. Thus, the protection of this vulnerable population must be an important component of public health measures.

**Disclosure:**

No significant relationships.

